# Using Hierarchical Bayes to Understand Movement, Health, and Survival in the Endangered North Atlantic Right Whale

**DOI:** 10.1371/journal.pone.0064166

**Published:** 2013-06-06

**Authors:** Robert S. Schick, Scott D. Kraus, Rosalind M. Rolland, Amy R. Knowlton, Philip K. Hamilton, Heather M. Pettis, Robert D. Kenney, James S. Clark

**Affiliations:** 1 Nicholas School of the Environment, Duke University, Durham, North Carolina, United States of America; 2 Centre for Research into Ecological and Environmental Modeling, University of St. Andrews, St. Andrews, Scotland, United Kingdom; 3 John H. Prescott Marine Laboratory, New England Aquarium, Boston, Massachusetts, United States of America; 4 Graduate School of Oceanography, University of Rhode Island, Narragansett, Rhode Island, United States of America; Institut Pluridisciplinaire Hubert Curien, France

## Abstract

Body condition is an indicator of health, and it plays a key role in many vital processes for mammalian species. While evidence of individual body condition can be obtained, these observations provide just brief glimpses into the health state of the animal. An analytical framework is needed for understanding how health of animals changes over space and time.Through knowledge of individual health we can better understand the status of populations. This is particularly important in endangered species, where the consequences of disruption of critical biological functions can push groups of animals rapidly toward extinction. Here we built a state-space model that provides estimates of movement, health, and survival. We assimilated 30+ years of photographic evidence of body condition and three additional visual health parameters in individual North Atlantic right whales, together with survey data, to infer the true health status as it changes over space and time. We also included the effect of reproductive status and entanglement status on health. At the population level, we estimated differential movement patterns in males and females. At the individual level, we estimated the likely animal locations each month. We estimated the relationship between observed and latent health status. Observations of body condition, skin condition, cyamid infestation on the blowholes, and rake marks all provided measures of the true underlying health. The resulting time series of individual health highlight both normal variations in health status and how anthropogenic stressors can affect the health and, ultimately, the survival of individuals. This modeling approach provides information for monitoring of health in right whales, as well as a framework for integrating observational data at the level of individuals up through the health status of the population. This framework can be broadly applied to a variety of systems – terrestrial and marine – where sporadic observations of individuals exist.

## Introduction

Long-term studies of body condition and survival of individual animals have provided critical insight into many different aspects of animal ecology [1], [2]. Though studies across a variety of taxa have shown how individual condition changes across time and space [3]–[7], there remain relatively few studies that can track changes in body condition at short time and space scales. Therefore ecologists are left trying to piece together the many likely paths that may connect the previous sighting with the current sighting. These paths could be spatial, i.e., where the animal was in the previous time periods. But they could also be physiological. For example, at an initial sighting an animal could have appeared healthy, but at a later sighting its health could have degraded markedly. The inability to reconstruct the pathway that leads to the current observed health state can hamper management, especially for species for which the survival of single individuals is of great importance [8]. Here we build a model to address two hidden processes in individual animals: changing health, and movement. We use estimates of these processes to infer individual survival.

Given a set of recapture or resighting histories, researchers typically use Cormack-Jolly-Seber models to address individual survival [9], [10]. Survival as a function of a time-varying covariate, e.g., body condition, can be included in models of this type [9], [11]. While these approaches have a long history in ecology, certain assumptions central to the modeling can be limiting. These include independence among individuals, and permanent emigration out of the study area [12]. Hierarchical Bayesian (HB) modeling [13] represents one potential approach to address several of these limitations. Alternate formulations for mark-recapture data allow for extensions that consider additional processes and individual heterogeneity [12], [14], [15]. Given a set of resighting histories along with ancillary observational data, we can use HB modeling to estimate hidden ecological processes of interest. These could include estimates of location [16] behavior [17], fecundity [18], body condition [19], and demographic status [15]. Given estimates of these ecological processes, we can then infer survival probabilities of individual animals as a function of those hidden processes. In addition, we can explore hypotheses, and, in certain systems, provide information to aid conservation and management decisions. This information could include the effect of anthropogenic stressors on health.

The endangered North Atlantic right whale provides an ideal study system to explore how individual health and movement affect survival. (In this paper “right whales” refers to the species found in the North Atlantic Ocean, *Eubalaena glacialis*.) Right whales remain one of the most endangered large whales [20], with current estimates of population size around 500 animals [21]. Because their habitat is one of the most heavily industrialized stretches of ocean [22], right whales are vulnerable to many anthropogenic impacts. Two of these are the leading causes of serious injury and mortality – ship strikes and entanglement in fishing gear [20], [23].

**Figure 1:**
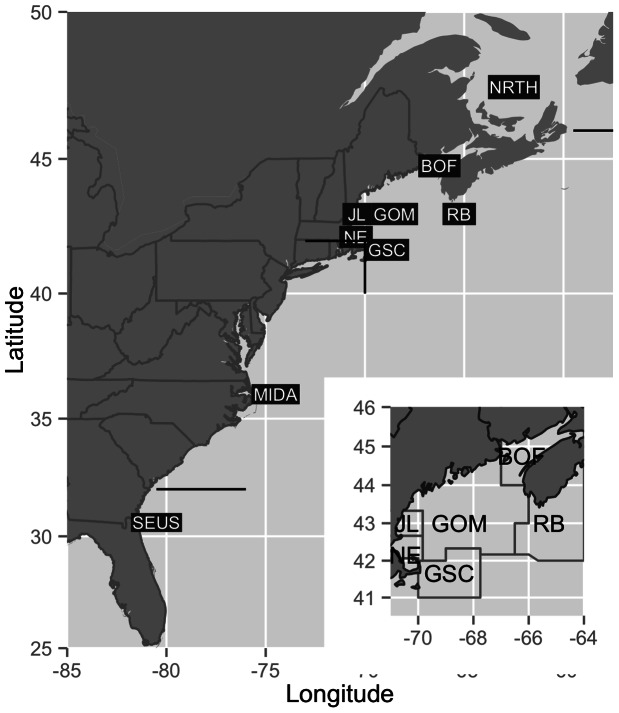
Overview map of right whale habitat. Overview map of the study area with the 9 geographic areas labeled at the approximate centroid for each region. Inset map highlights the regions contained within the greater Gulf of Maine. Abbreviations are as follows: NRTH  =  North region, BOF  =  Bay of Fundy, JL  =  Jeffreys Ledge, GOM  =  Gulf of Maine, RB  =  Roseway Basin, NE  =  Northeast, GSC  =  Great South Channel, MIDA  =  Mid-Atlantic, and SEUS  =  Southeastern US.

Photographic mark-recapture studies of right whales throughout their range have led to a broad-scale understanding of movement patterns and health status at both the population and individual levels [24]–[28]. The primary seasonal habitats for right whales and the movements between these habitats are broadly understood from individual observations made throughout the western North Atlantic over the past 30+ years [22], [29], [30]. While the macro-scale movement patterns are known, there is a great deal of individual variability. Most adult females who are reproductively active are seen with calves in the southeastern United States (SEUS), Cape Cod Bay (CCB), and the Bay of Fundy (BOF) [24] (Figure 1). However, there is a significant subset of these females who rarely or never take their calves to the BOF [31]. In addition, evidence from satellite telemetry studies suggests large individual variation in movement patterns over their entire habitat [32], [33]. Further, all females are rarely sighted in the year immediately following calving [34]. Despite many years of study, these individual differences in sightability and habitat use complicate our understanding of the dynamics of the population, and these differences can make spatially explicit management strategies difficult to implement [35].

From the photographic evidence, we have documented that right whale body condition can change over time and varies with the female reproductive cycle [36]–[38]. While there is a great deal of individual variability, larger-scale patterns have emerged from the photographic observation data. Whales that are scored in the poorest body condition typically die or are never seen again [36]. Body condition studies have also documented how the physiologic stressors faced by reproductively active females are quite different from those of adult males [36], [39]. Given that observation frequencies of individuals vary within and across years, it is difficult to assess both the changes in body condition, as well as the causes of those changes. Understanding the individual and population-level health status could provide critical information for the management of this species during periods of natural stress, e.g., periods of food limitation [40], as well as during periods of anthropogenic stress, e.g., oil and gas exploration, marine renewable development.

**Figure 2:**
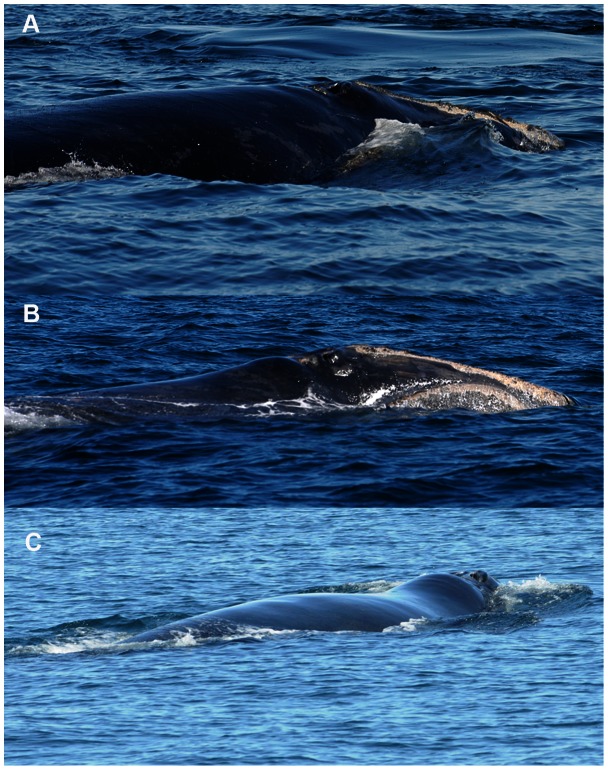
Body condition photos. Plate showing examples of the different classes of body condition judged by the evaluation of the dorsal back profile in the post-blowhole region: a) good, b) fair, and c) poor. Note the contrast between the level to convex nuchal area in a) and the concavity where the back drops off behind a pronounced hump in c). Photographs were taken under permits from the National Marine Fisheries Service (#15415) and the Department of Fisheries and Oceans, Canada. Photo credit: New England Aquarium.

**Figure 3:**
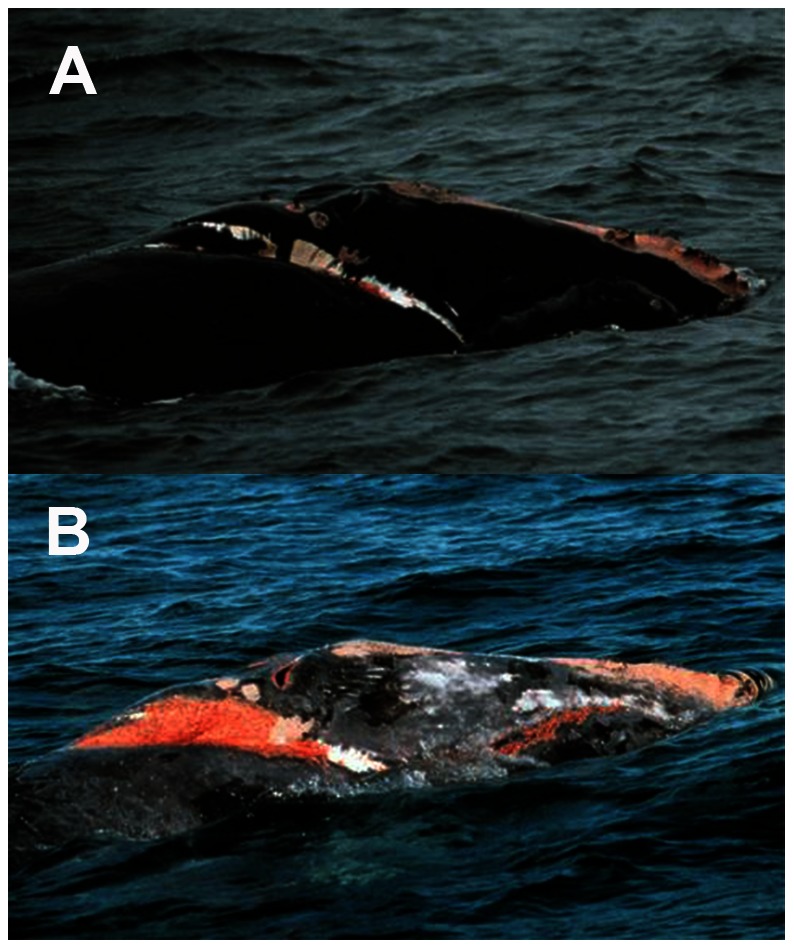
Visual health parameters. Photo plate depicting whale #1621, an adult male last seen in 2000. Photo in panel a) was taken in 1997, and panel b) was taken in 1999. Photos show examples from four of the visual health parameters: 1) entanglement severity, 2) presence of orange cyamids around the blowholes, 3) poor skin condition, and 4) rake marks forward of the blowholes. There are multiple ordinal classes within each health parameter. For example, in a) skin condition received a score of 2– good skin condition, while in b) skin condition would receive a score of 1– poor skin condition.

Here we build a HB model that assimilates 30+ years of data in an effort to understand how the health and movement of individual animals in different demographic categories change over time. We incorporate long-term broadscale survey information [37], [41], [42], and 4 visual health parameters [36] to estimate the true, but hidden, health of individual animals. These parameters include body condition (Figure 2), skin condition, the presence of cyamids (“whale lice”) around the blowholes, and the presence of rake marks forward of the blowholes (Figure 3). We also include the reproductive status of adult females, and visual estimates of entanglement severity. We use location data from documented sightings and prior knowledge to estimate movements of individuals in broad geographic regions at a monthly time step. Using this approach we are also able to estimate the health status and movements of individuals. In turn we use these estimates to quantify survival at both individual and population levels. These estimates identify the changing patterns of the health status in this population, as well as create a framework for understanding the effects of anthropogenic stressors.

## Methods

**Figure 4:**
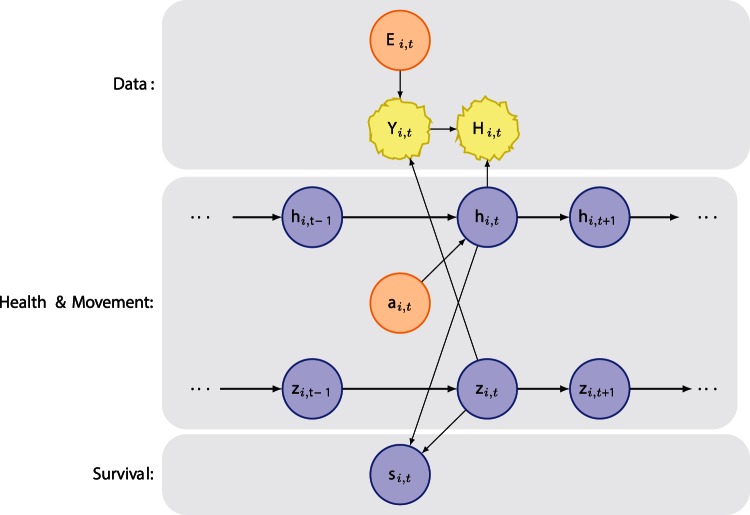
Graphical depiction of the statistical model. Graphical model depicting the dependency structure. We have observation models for the visual health parameters *H*, for survey effort *E*, and for sightings *Y* (top panel). The middle panel comprises two process models for the latent states of health *h*, and movement, *z*. Lastly, survival, *s*, is estimated as a function of latent health and movement.

To make inference on the movement patterns, relative health status, and ultimately survival of individuals, we built a hierarchical Bayesian model. The temporal resolution of the model is monthly dating from 1980 to present; the spatial resolution is at the level of the primary geographic regions that comprise right whale habitat (Figure 1). The model assimilates aerial and vessel survey information, locations of identified individual right whales, photographic evidence of health status across several health parameters comprised of ordinal classes, and prior knowledge. The three process models in the main model provide inference on movement, health, and survival of individual right whales (Figure 4). Class, or population-level, summaries can be inferred.

### Data

We included three primary sources of data in the model: 1) spatially and temporally explicit survey effort; 2) sightings of individuals; and 3) photographs that accompanied the sightings. The survey methods are described in detail in [42], but are summarized briefly here. Surveys are conducted from a boat or plane. The survey data contain spatial and temporal information, i.e., the spatial position of the boat or plane through time, as well as environmental data (e.g., visibility, Beaufort sea state). Survey data extend from November of 1978 to the present, and were obtained from the North Atlantic Right Whale Consortium [43]. We aggregated individual surveys, regardless of the platform, to regional summaries at a monthly time step. Survey effort is summarized by the primary geographic regions that comprise right whale habitat (Figure 1). We summarized the data by summing the length of all on-effort survey tracks within each region during each month of analysis (km/region/month), where on-effort was defined as any segment of survey track where one or more observers were on watch, visibility was at least 2 nautical miles (3.7 km), and sea state was no higher than Beaufort 3.

Sightings data, both on-survey and opportunistic, were also obtained from the NARWC, and though they extend back to the 1930′s, the bulk of the data are from 1980 onward. The sightings data consist of unique sighting records for each individual, and include information on the individual ID, date, time, and location of sighting. (N.B. EGNo is the unique identifier for individuals in the New England Aquarium's right whale database http://rwcatalog.neaq.org/, last accessed 10 October 2012. Hereafter, the # followed by a 4 digit number will refer to that specific whale.) Ancillary data can include behavioral information, age, gender, and calving history for females. Whales can be sighted from 0 to many times each month, and it is these individual records that comprise the sightings information in the model (Figure 4).

Each sighting is accompanied by at least one photograph, which is used to match the individual whale to the existing catalog. However, typically a full suite of photographs from head to flukes are taken of each individual. Visual health information is collected from these photographic suites, and one score for each category is assigned to the animal in a given month [36], [39]. In addition to measures of body condition (Figure 2), researchers have documented three additional visual health parameters: 1) the presence of cyamids around the blowholes; 2) the presence of rake marks forward of the blowholes; and 3) indices of the skin condition of the individual [36], [39] (Figure 3). All of these 4 visual health parameters are comprised of different classes on an ordinal scale and are the observations that provide inference on true health.

We include two other health parameters in the model, one of which stems from the photographic observations of individual whales. Both the entanglement status and entanglement injury severity of an individual whale can be observed [23]. Here we defined three ordinal classes: 1) moderate entanglement injuries with gear attached and severe entanglement injuries with or without gear; 2) minor & moderate entanglement injuries without attached gear; and 3) non-entangled. Lastly, for mature females, we include calving status as a contributing factor to health under the assumption that gestating animals will be in better body condition than lactating females [36], [38], [39], [44].

Below we summarize the main components of the model. See Appendix S1 for more detail on model structure and computation.

### Model Summary

Consider a live whale *i* at month *t* occupying a zone 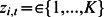 characterized by age *a_i,t_* and health status *h_i,t_* >0. Here “zone” refers to a geographical region as defined above (Figure 1). A live whale thus is defined by state vector 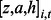. On rare occasions an individual is sighted in more than one region within a given month, in which case *z_i,t_* is the first location in which the animal was sighted. There were 631 such events, out of 96,099 possible individual-month combinations, or 0.66%. Depending on its health status and differential mortality risk posed by, for example, vessel traffic that could differ between zones *k*, the individual survives (*s_ik,t_*  = 1) to month *t*+1 with probability 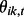. During this month (*t*, *t*+1) it may remain in *k* with probability *m_kk_* or move to zone *l* with probability *m_lk_*. Using the sightings and the visual health parameters, we wish to infer the effects of age and previous health status on current health as well as the effect of health and the differential risk associated with zones on survival. This formulation allows us to test if geographic zones with higher levels of human activity, e.g., vessel traffic and/or fishing gear, may be worse for right whale health. The time of death *T_i_* is typically unknown, but is known in some cases. When time of death is known, age at death *A_i_*_,_ could be known, if year of birth is known. Month *t* is the number of months since the beginning of modeling, January 1970, and *t* ^(*μ*)^ is the month *μ* = 1,…,12 to which time *t* belongs.

### Data models

#### Sightings

The number of sightings of individual *i* in zone *k* in month *t* is
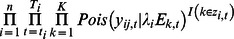



where *t_i_* and *T_i_* are the imputed birth and death months, *z_i,t_* is the location of *i* in month *t*, *E_k,t_* is the search effort (km) in zone *k* in month *t*, and the 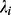 is the expected number of sightings per unit effort for individual *i*. *z_i,t_* is known for individuals and months where there are sightings (*y_ik,t_* >0), and it is imputed for other individuals and months.

### Ordinal Health Classes

For each of 4 health parameters, the observed relative health of an individual whale is classified on an ordinal scale. This scale is related to underlying relative health status in a non-linear fashion. The synthesis of the different types of health parameters requires a standard latent scale that allows for indirect integration. We can view this latent scale as a representation of the true health status of the animal that is responsible for different health parameters, each of which might be represented by observations taken on an ordinal scale. Each discrete class of a health parameter is viewed as the consequence of health state, but then is observed with error. We use a multinomial logit, each dependent on this same latent scale as basis for synthesis.

Status observations of type *q* are ordinal on discrete space 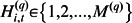 where health score 1 indicates poor health and *M* ^(*q*)^ indicates best health. The six types of observations used in this analysis include: 1) indices of body condition; 2) scarring severity from entanglement in fishing gear; 3) skin condition; 4) the presence of rake marks forward of the blowholes; 5) cyamids around the blowholes; and 6) reproductive status. The numbers of discrete ordinal health classes for each health parameter range from 2 to 3. For example, body condition is comprised of three classes: 1 (poor), 2 (fair), and 3 (good). For skin condition the classes are 1 (poor skin condition) and 2 (good skin condition). The latent health index *h_i,t_* is defined on the arbitrary scale (0, 100). Consider an example for one health parameter with three classes. The observation model is
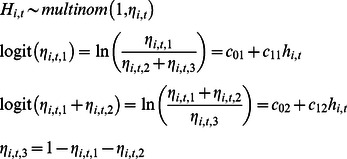



where there is a vector of probabilities associated with each of the health classes 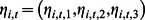 and four fitted coefficients  
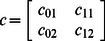
  that translate the continuous scale for *h* to the ordinal scale for *H*. The coefficients are ordered such that 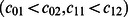.

The latent health scale facilitates prior specification. We define breakpoints on the *h* scale, i.e., values of *h* defining change from state *H*  =  *k*–1 to *H*  =  *k* at probability  = ½. The prior on breakpoints is flat over non-overlapping ranges
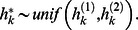



The gradient at a transition *k* is largely determined by the slope parameters *c*_1*k*_. The priors for the breakpoints for each visual health parameter are in Figure S1 of Appendix S1.

### Process model

#### Health status

Individual health is a latent state
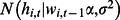



where *α* is the vector of fitted coefficients, and the design vector 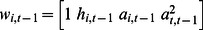 includes an intercept, an AR(1) term for temporal coherence in health, and age terms to allow for that fact that survival probabilities initially increase 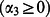 but can eventually decline with age 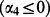. There is process error 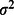. The prior parameter values are 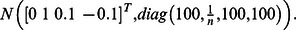


#### Survival

The probability of survival from *t* to *t* +1 is 
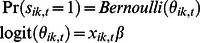


where the design vector contains health status and a fixed effect for the zone. The prior is non-informative for zone. Animals not seen after 6 years are presumed dead [45].

#### Movement

Location is sometimes known and sometimes imputed. Let *z_i,t_* indicate a zone occupied by *i* in month *t* and 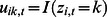 be the occupancy vector indicating the event that *i* is in *k* at *t*. If the individual is observed in *k* at *t*, then 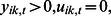 and 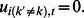 The individual may also be in *k* at times when it is not observed. The event of moving from *j* to *k* at some time during month *t* (between *t* and *t*+1) is
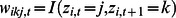



The probability associated with this event is


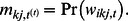
 Note that 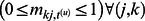 and 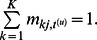


We set informed priors on the relationship between true health and health observations, and on movement (Figure S2 in Appendix S1). Using the sightings data, we fit the model to data in a Gibbs sampling framework [13]. We ran the model for 200,000 iterations, checking for convergence of the chains, and discarding the first 50,000 iterations as burn-in. Convergence of the chains was assessed visually.

## Results

### Movement

**Figure 5:**
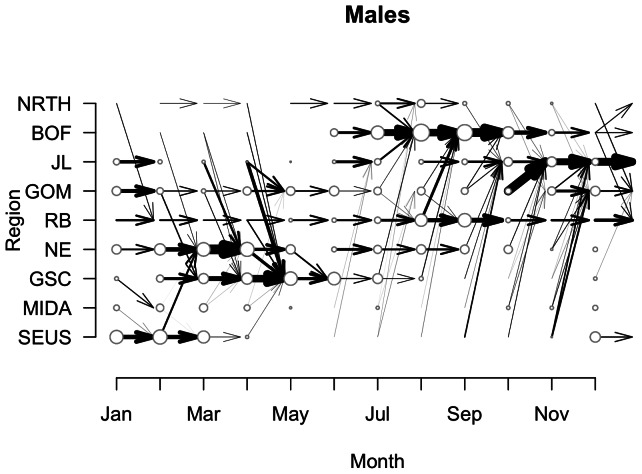
Movement transitions for male right whales. Posterior estimates of transitions made by male right whales between regions over the course of the year. Size of the circles in each region at each month correspond to the actual number of male right whales observed. Lines connecting regions indicate probability of transition, *p_jkt_* >0.25. Magnitude of probability is depicted by line thickness.

**Figure 6:**
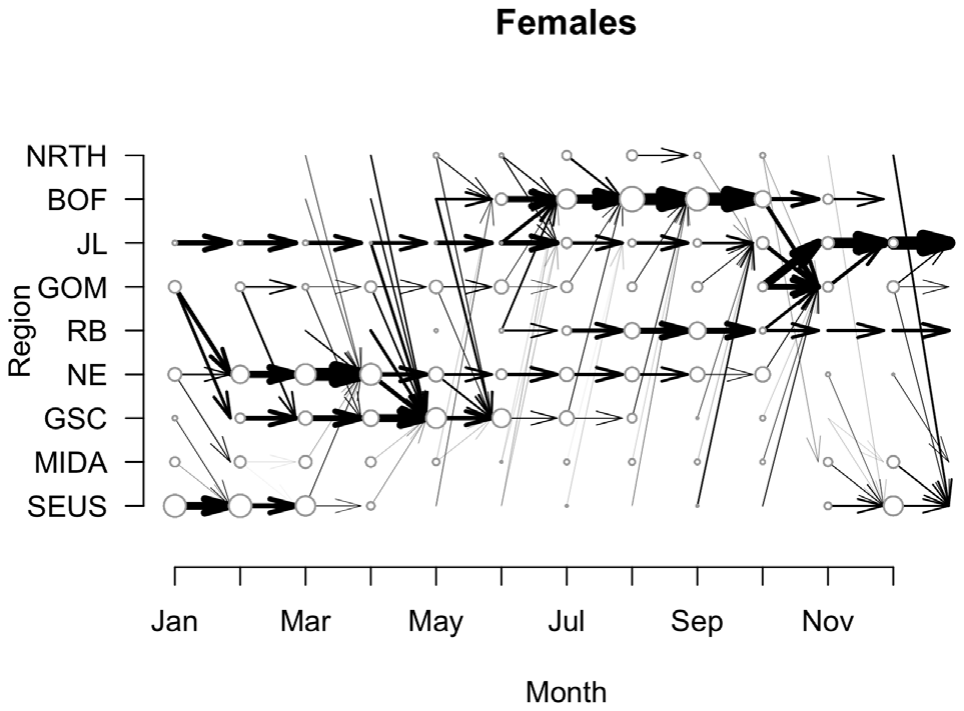
Movement transitions for female right whales. Posterior estimates of transitions made by female right whales between regions over the course of the year. Lines and circles as in Figure 5. In contrast to males, females spend more time in BOF, and have more estimated transitions to SEUS at the end of the year.

At the population level, we have estimated the paths between regions taken by adult males (Figure 5), adult females (Figure 6), and unknown gender adults (results not shown). Broadly speaking, males and females have relatively similar estimates of movement transitions (Figures 5 & 6). That is, for males and females, movement into and out of the broad geographic regions occurs at approximately the same time (Figures 5 & 6). However, the relative importance of each region, as defined by the duration and timing of entry/exit patterns, differs as a function of gender in certain key areas. For example, the Bay of Fundy (BOF) is one of the main habitat regions for right whales, yet it appears it is more important for females (Figure 6). Note that both in terms of duration in the BOF, and movement to the BOF, females move there earlier and stay longer than males, and more transitions out of BOF are estimated (Figures 5 & 6). In contrast more transitions to the GOM and JL late in the year are estimated for males than females. Females are more likely to move to the SEUS than males in November and December. In addition, we see more estimates of transitions through MIDA for females than for males.

While we used informed priors for the movement transitions, it is clear that a) certain little-known or presumed transitions have emerged as important, and b) certain presumed transitions are rarely observed. For the first case, in males, we had a strong prior on movement from GOM to BOF in July and weak priors for the transitions from Jeffreys Ledge (JL), Great South Channel (GSC), and BOF to BOF (Appendix S1). However, the latter three movement probability estimates were higher than the former (Figure 5). For the second case, in females, we had a strong prior on movements from the southeastern US (SEUS) to the Mid-Atlantic region (MIDA) and to the Northeast region (NE) in the early part of the year, as right whales observed in SEUS and later in NE, must make the transition through MIDA. However, estimates of the transition probabilities through MIDA were relatively low (Figure 6), and biased towards the southward migration. For males there are relatively few estimated transitions through MIDA (Figures 5 & 6).

**Figure 7:**
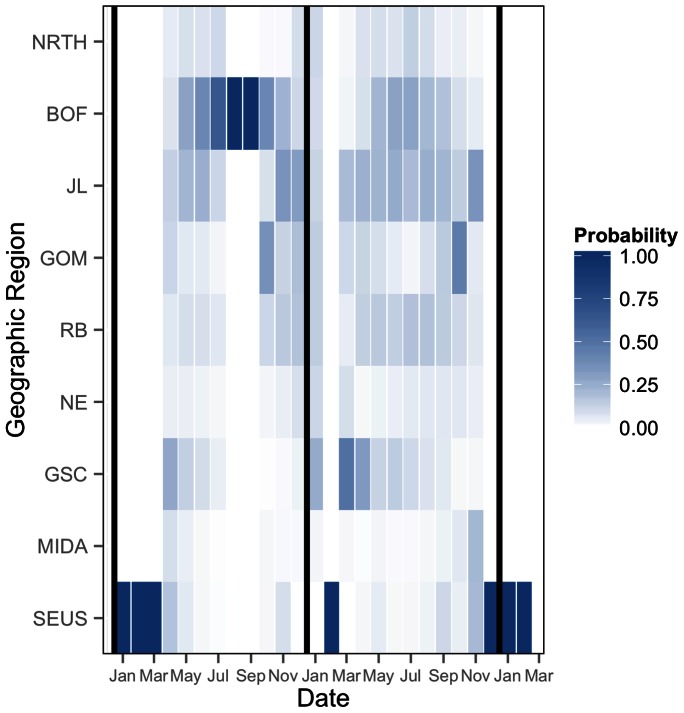
Movement transitions for whale #3911. Heat map depicting Pr(*z_i,t_*  =  *k*) for the entire sighting record of #3911. Darker blue represents higher probability; darkest blue indicates animal was sighted in that region, i.e., #3911 was observed in August and September of 2009 in BOF. In the latter half of 2010, probable transitions are from BOF to GOM to MIDA to SEUS where the animal was observed in December. Vertical black lines indicate the start of 2009, 2010, and 2011.

At the individual level, we can use estimates of *z_ijk,t_* to examine likely movement paths through the geographic regions. Though whale #3911 had a very short sightings record, and was only seen in two regions (SEUS and BOF), we can use estimates of movement transitions to reconstruct likely movement paths. In particular, between her first and last sightings in SEUS in February and December 2010, #3911 likely moved through GSC, BOF, JL, and MIDA (Figure 7). By combining posterior estimates of health with estimates of movement, we can begin to discern movement transitions that could be injurious to individual whales. For example, #3911 had a severe entanglement just prior to her death that resulted in a rapid decline in health.

### Health

**Figure 8:**
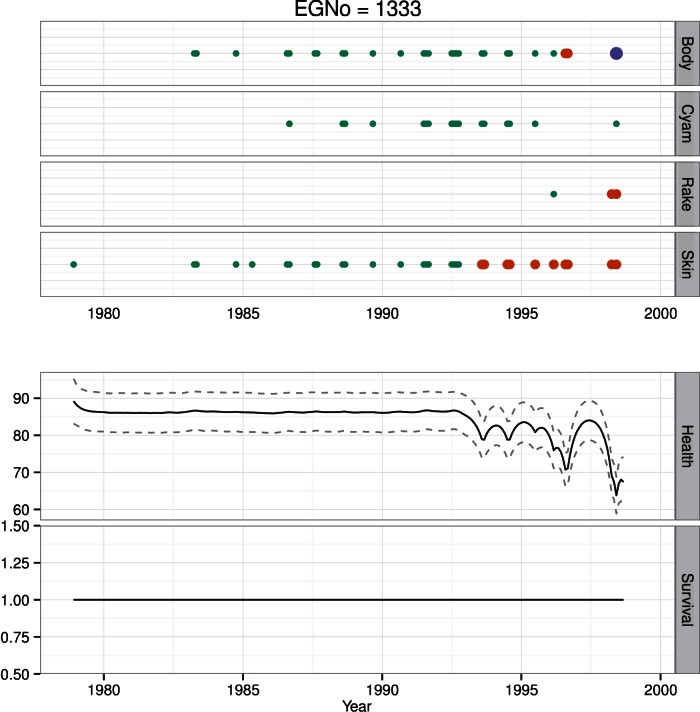
Health time series for whale #1333. Time series of health observations *H* for skin condition, body condition, cyamids and rake marks (circles), estimates with uncertainty of health *h* (thick line and dashed lines), and estimates of survival *s* (height rectangle at bottom) for #1333. Photographic observations of *H* are color and size coded by class. For a visual health parameter with three categories, e.g., body condition, green is the best category, orange is fair, and purple is poor. For a visual health parameter with two categories, e.g., skin condition, green is the best category, while orange is poor. #1333 had a gradual decline in health over a period of years, and was observed dead in 1998.

**Figure 9:**
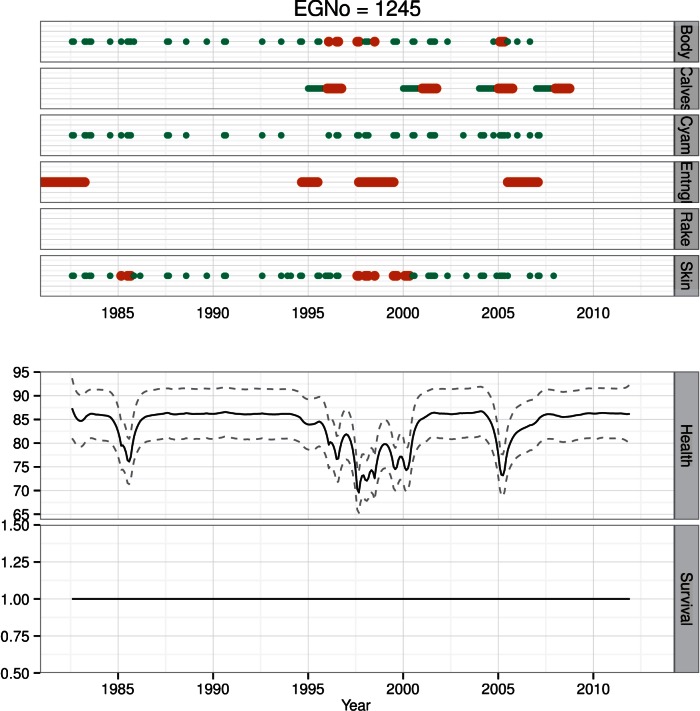
Health time series for whale #1245. Photographic observations of *H* are color and size coded as in Figure 8. Additional health observations *H*, include periods of entanglement, and calving status. #1245 has experienced several periods of compromised health, but has recovered from each, and is currently imputed to be alive.

**Figure 10:**
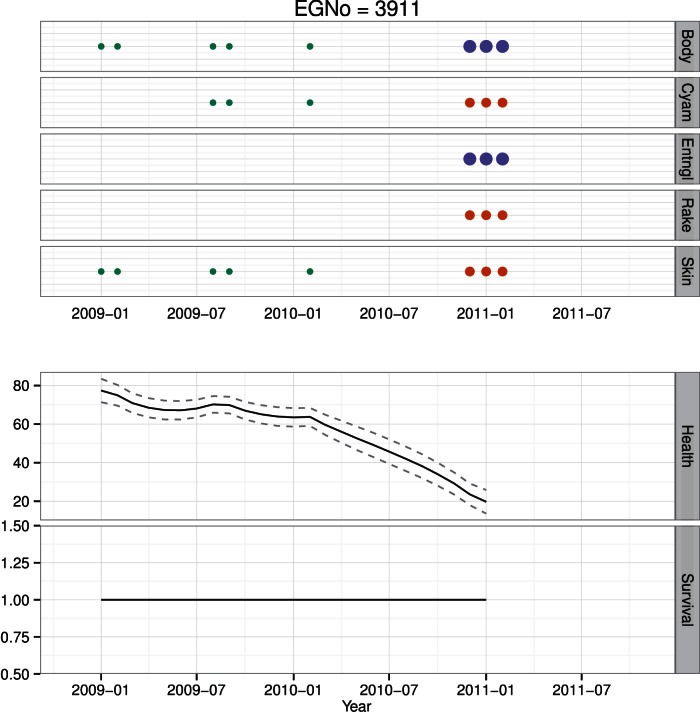
Health time series for whale #3911. Photographic observations of *H* are color and size coded as in Figure 8. #3911 was only alive briefly, had declining health towards the end her life as a result of a severe entanglement, and was observed dead in February of 2011.

**Figure 11:**
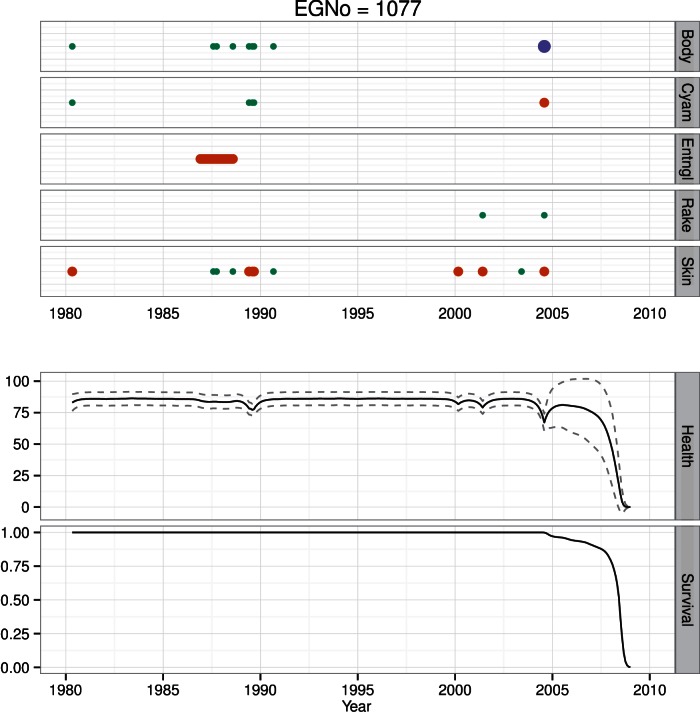
Health time series for whale #1077. #1077 has not been seen since 2004. At the time of the last sighting its health status was poor. Estimates of health since the last sighting have declined to 0, and the animal is currently presumed to be dead.

At the population level, health is positively linked to health at the previous time, as well as weakly positively and negatively linked to linear and quadratic terms for age (Table 1). We chose four individuals to illustrate the output from the model, because these individuals exhibited a broad range of health trajectories. These included: 1) an adult male (#1333) with a multi-year decline in health followed by a known death (Figure 8); 2) a reproductively active female (#1245) with a long and detailed sighting history (Figure 9); 3) a juvenile female (#3911) with a short sighting history and a severe entanglement leading to death (Figure 10); and 4) a rarely seen adult male (#1077) with a sparse sighting history (Figure 11).

**Table 1:** Estimates of the beta parameters in the regression for health, *h_t_*. Health at the previous time-step has a strong relationship to health at current time-step.

Term	Mean	2.5%	97.5%
Intercept	−0.78	−1.9	0.33
*h_t-1_*	0.99	0.96	1.02
Age	0.08	0	0.41
Age^2^	0	−0.01	0

Health is weakly related to age, but not to Age^2^.

Whale #1333 was first seen in 1978, and was last seen dead on a beach in Virginia in 1998. For the first 15 years the animal was in good condition both in terms of skin and body condition (Figure 8). In addition, there was no external evidence of anthropogenic impacts. In the early 1990′s, however, his skin condition deteriorated, while his body condition remained good. In the late 1990′s his body condition worsened. Finally the animal died of unknown causes in October of 1998. The model quantifies the trajectory of this decline, and provides an estimate of uncertainty around the health estimates. When photographic evidence indicated that #1333 was healthy, there is more uncertainty in the estimate. In contrast, when the animal had poor skin and body condition, the uncertainty around the estimates narrows considerably (Figure 8).

Whale #1245 was first seen in 1982, and last seen in 2010. Observations of her health are numerous and they have varied widely; she has been involved in at least 4 minor entanglement events, each lasting for various durations (Figure 9). In addition, she has birthed at least 4 calves (Figure 9). Save for a brief unhealthy period in the mid-1980′s when she was a juvenile, #1245 was healthy in her pre-reproductive years (Figure 9). However, once she began calving (1996) her health declined for a period of approximately 5 years before recovering in the early 2000′s. Whale #1245 had a longer than average inter-calf interval following her first calf in 1996, and since then has produced three more calves. While estimates of her true health have increased since the low period in the late 1990′s, there has also been much more variability compared to her pre-reproductive years. This variability is likely due in part to the natural physiological demands placed on reproductively active females [36], [44]. These demands manifest themselves as a visible reduction in body condition [36], [38].

Whale #3911 lived just briefly, dying at age 2 as a result of weight loss and cachexia from a chronic entanglement [46] (Figure 10). She was seen in the SEUS in February 2010 in good health, and then was resighted in the SEUS in December 2010 severely entangled and in very poor condition [46]. Whale #3911′s condition deteriorated quickly once entangled (Figure 10). Though we do not know where or when #3911 became entangled, we have an estimate of her movements between February and December (Figure 7), suggesting that #3911 spent significant time, in chronological order, in the GSC, JL, BOF and GOM before returning via JL to SEUS (Figure 7).

Individual #1077 is an adult male with a relatively sparse sighting history over 25 years (Figure 11). The animal was last seen in poor condition in August of 2004. #1077 was sighted in poor skin condition a few times in the 1980′s, but was not seen for much of the 1990′s. In the 2000′s, #1077 was again seen with poor skin condition, and was subsequently sighted in both poor skin condition and poor body condition (like #1333). Following the last sighting, estimates of his health decreased rapidly (Figure 11).

### Survival

We estimated individual survival for every animal in the North Atlantic Right Whale Catalog. At the population level we found a positive link between health and survival, but no differential effect of region on survival (Table 2). Here we use the same four animals to illustrate different survival patterns present in the population. Whale #1333 had a constant survival probability up until his known death in October 1998. While his health was clearly in decline, estimates of survival were still high through the end of his life (Figure 8). The health estimates for female #1245 were not as low as #1333′s, and she is currently estimated to be alive (Figure 9). Like #1333, #3911 has a constant survival probability up through her death (Figure 10). Though this may seem counterintuitive, since both of these animals were seen alive immediately prior to their deaths, survival is imputed to be 1 for these preceding months. Like #3911, #1077 was in poor health at the time of its last sighting, however in contrast to both #3911 and #1333, #1077 was not observed dead. In this case #1077 is now presumed dead, but has a declining survival probability following its last sighting (Figure 11).

**Table 2:** Estimates for the parameters for survival as a function of health and location.

Term	Mean	2.5%	97.5%
Health	0.13	0.08	0.19
BOF	−0.001	−0.0009	0
GOM	−0.0003	−0.0009	0
GSC	−0.0003	−0.0009	0
JL	−0.0003	−0.001	0
MIDA	−0.0002	−0.001	0
NE	−0.002	−0.008	0
NRTH	−0.0005	−0.001	0
RB	−0. 0004	−0.001	0
SEUS	0	−0.0003	0

There is a positive slope (Health), but no significant relationships between region and survival.

Whales #1077 and #1333 represent an interesting point of comparison. They are both animals with long sighting histories; however they contrasted in that one had a sparse sighting history and is presumed dead (#1077) while the other had a detailed sighting history and is known to be dead (#1333). Whale #1077 was in poor condition at its last sighting, as was #1333 (Figures 8 & 11). The difference in the survival estimates for each of these animals is that #1333 was observed dead, while #1077 was never seen again and its survival probability decays over time in parallel with the decrease in health.

### Links Between Observed Health and Latent Health

**Figure 12:**
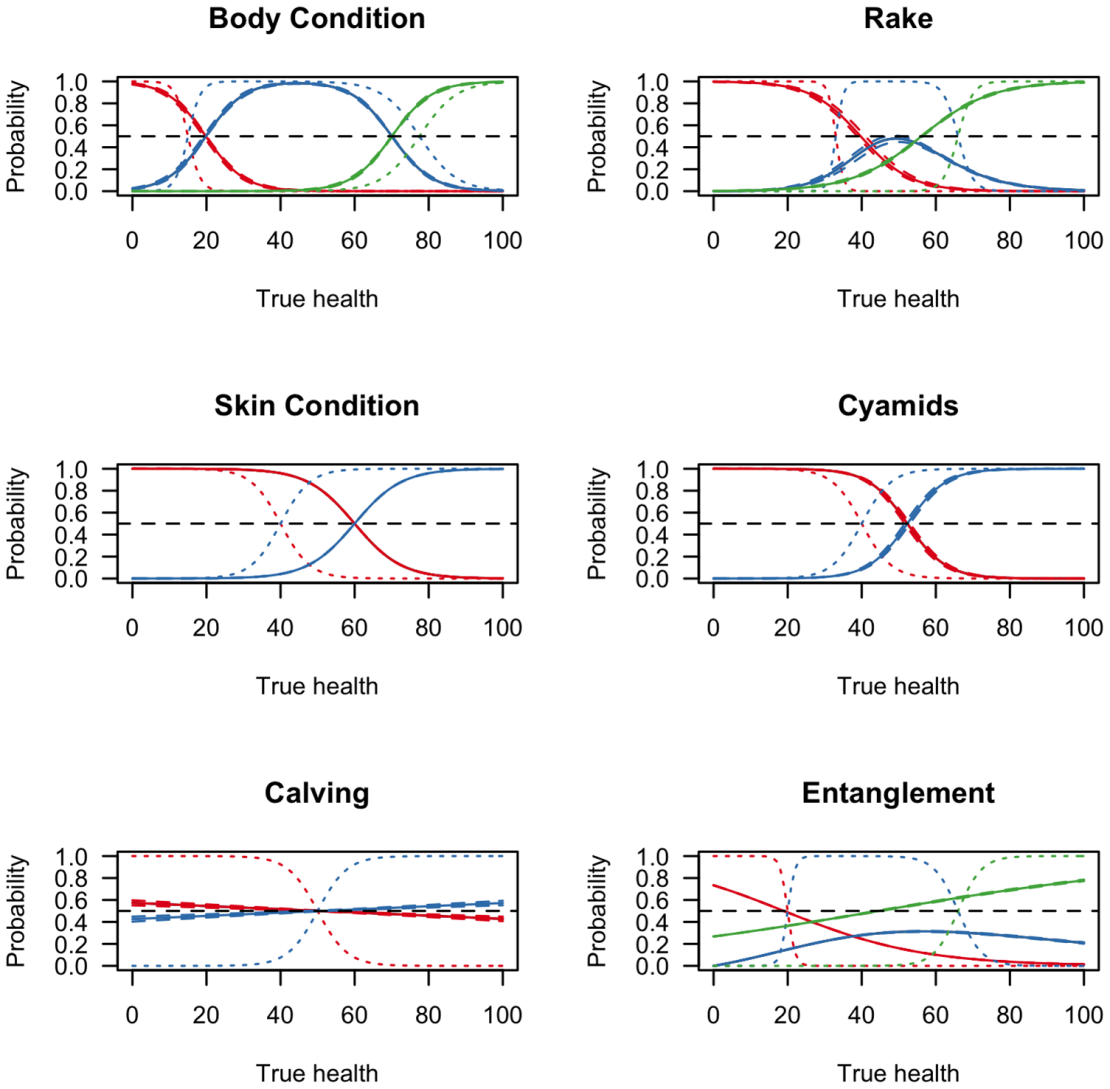
Relationship between *h* and *H*. Graphical representation of the parameters in matrix c, which relate observed health *H* to true health *h*. The solid curves represent the estimated probability with uncertainty (dashed lines) that given an estimate of true health *h*, the animal would be seen in a given health class for each of the 6 health parameters. Priors are depicted with dotted lines. Estimates for the parameters governing body condition, rake marks, skin condition, and cyamids show a clear relationship between true health status and observed health. In contrast, the relationship between calving and entanglement and true health is not clear.

The links between observations *H* and underlying health *h* are most clearly defined for body condition, skin condition, presence of rake marks, and presence of cyamids (Figure 12). In contrast, the estimates for calving and entanglement do not provide clear relationships between an observation and the true underlying health status.

For body condition, results suggest that the break between class 1 (poor condition) and class 2 (fair condition) is occurring at higher health levels. We presumed a very low health value for this break point, when in fact the shift appears to occur at higher levels and more gradually than we presumed (Figure 12). In contrast, the shift between the fair and good categories appears to come at a lower health level than we presumed. For skin condition and for cyamid infestation, the posterior estimates of the breaks between the two classes are shifted higher than the prior (Figure 12). While there is a clear link between these visual health parameters and the underlying health, these results suggest that the change from one class to another occurs at higher levels of health than initially presumed.

We did not recover significant parameter estimates, i.e. non-zero, for the link between calving status and health (Figure 12). This was a surprising result for calving status, but see Discussion. For entanglement injuries, animals with severe entanglements (class 1) had a higher probability of being in poor health; whales in this class were moderately entangled and carrying gear or severely entangled with and without gear. For animals in the middle class, i.e., minor and moderate entanglement without gear, or in the non-entangled class, the link between observations and health was less clear. This means a severely entangled whale is most likely in poor health. In contrast, the other two classes of entanglement are less likely to indicate poor health.

For the presence of rake marks, the shape of the response is similar to that for body condition, however, the distinction between classes is not clear (Figure 12). Specifically, in the middle class (blue line), the peak probability is around *h* = 50, but there is also high probability at this same point of being in either class 1 or class 3. This means that observations of animals in class 1 or 3 provide clear evidence of being in poor or good health, respectively.

## Discussion

Observations of right whales are unequally distributed both across their known habitats and through time [22]. Because of this, inference on where the animals go and how their health changes during transition through these areas is difficult. Here we tied together sightings and effort data to provide individual (Figure 7) and population-level estimates of movement states and movement transitions (Figures 5 & 6). We have quantified how individual health a) decays through time (Figure 8), b) differs between unknown and known death status (Figures 10 & 11), and c) drastically declines following severe entanglement (Figure 10). Additionally, we have shown how well the photographic observations of different health parameters represent the true health status of individuals (Figure 12). These advances provide a better understanding of health at the individual and population levels, and will allow for future explorations of how various aspects of right whale life history impact health. In addition, it provides a framework for understanding the impact of different anthropogenic stressors on right whale health and survival that may be used to develop more targeted mitigation strategies.

### Movement

The population-level estimates of movement transitions generally support current understanding of how right whales move through their habitats, and also highlight areas where more data are needed. From known transitions, the model estimates the differences between male and female movements out of the northeastern habitat area into SEUS (Figures 5 & 6). Females are estimated to transition to the SEUS from several regions at the end of the year (Figure 6). In contrast, males are more likely to transition out of the BOF into JL or the GOM (Figure 5). The model also estimates some females – likely non-pregnant females – moving to JL and GOM instead of SEUS (Figure 6). With respect to areas of research need, for both males and females, the mid-Atlantic region is an area where more survey effort is needed. Whales have been observed there throughout the year (Figures 5 & 6), yet relatively few are observed making the transition from northern habitats through the MIDA to the SEUS, or vice-versa. In particular, females have been sighted in the MIDA in each month of the year save for July (Figure 6), and this is the most vulnerable segment of the population [8]. As they transit through this region (MIDA) and all regions from BOF to SEUS, they swim past many large shipping ports and are vulnerable to ship strikes [35], [47]–[49]. More research effort is needed to quantify when and where they are moving in and out of this area. And more conservation intervention may be needed to protect whales in and around areas of high shipping traffic [35], [50], [51].

### Health

The photographic observations of health have provided a unique opportunity to build a state-space model, which allows for inference on the true underlying health status of the animal. We have successfully fit this model to data, and shown how health changes over time and space using four individual whale cases. This synoptic view of health is very powerful, because it provides a picture of the animal's health during times and places that the animal was not observed.

One of the most important results is our ability to estimate the decay and recovery in health. For example, #1077 had a relatively sparse sighting history, and its condition at the time of last sighting was poor (Figure 11). Model estimates of the decline in health prior to this last sighting are precise, but are relatively uncertain in the period following the last sighting. The animal was in good body condition in 1992, but had a nearly 10-year sighting gap. At its last sighting in 2004, it was in poor body condition, and it has not been seen since. In contrast #1245 has had many periods of apparently diminished health status, but recovered from all of them (Figure 9). Some of the intervals of poorer health are likely due to decreased body condition as a result of lactation, e.g., 1997 and 2006, while others are not, e.g., late 1990′s. Finally, in #1033, we estimated a slow decline in health as evidenced by poor skin condition in the mid-1990′s, but rapid declines for the periods of poorer body condition.

The four animals depicted here have varied sighting histories, and each highlights a different aspect of health and survival. In particular the difference between undocumented and known deaths provides an interesting contrast. For example, #1077 is presumed dead, and its estimate of declining health is broad, and survival probability initially declines slowly before declining rapidly to 0 (Figure 11). This contrasts with estimates of survival for the animals with documented deaths (Figures 8 & 10). The periods of extended poor health may precipitate some state change in the animal. With #1245, her extended poor health in the late 1990′s may have caused her longer inter-calf interval; this condition-mediated alteration in vital rates has been observed across a wide variety of taxa [6], [52], [53]. Similarly, in the mid- to late 1990′s, #1333 may have undergone a state transformation from healthy to sick – thereby making it more vulnerable to anthropogenic stressors (Figure 8).

Finally, estimates of the *c* parameters linking discrete photographic observations of health with true underlying health provide a clearer understanding of how well photographic observations indicate health status of the animal. Observations of body condition, skin condition, cyamid infestation, and rake marks are clearly linked to the health of the animal (Figure 12). Conversely, neither entanglement nor calving status is clearly linked to underlying health for all classes within each health parameter. As mentioned previously, the result for calving status was surprising, but we suspect two factors contribute to this result. First, fluctuations in body condition of gestating and lactating females are likely subsumed in the visual health parameter for body condition. Second, pregnant females are seen less frequently than females with a calf [34], and the relatively fewer sightings may make inference on this parameter difficult. For entanglement injuries, we suspect that there are issues related to the time-scale of observation, and are currently investigating alternate formulations for this parameter (See Future Work below). What the positive links do provide is the probability that an animal with a given true health status will be observed in a particular ordinal class for each health parameter.

### Future Work

There are several natural extensions to this work, including further work with entanglement and vessel strikes and movement, as well as examining population-level vital rates as a function of health. Exploring these will allow for a more comprehensive understanding of the factors affecting right whale health and survival.

Though we have included entanglement status here as a health category, we have only done so in a very preliminary fashion. Currently, there are 6 different entanglement classes in the data plus one for the non-entangled state, yet we have collapsed them to 3 classes for the initial application of the model to data (Figure 12). The collapsing was done in an ad hoc fashion, and though it represents a parsimonious initial attempt, it is possible that a more rigorous approach to classifying the levels of entanglement will more accurately reflect the impact of the injury on the whale's health. In many cases (*n* = 306) the animals have been entangled multiple times [23], and it is possible that regardless of severity, subsequent entanglements have cumulative effects on health.

Here we have shown initial glimpses into the movement of different population classes (Figures 5 & 6). A logical next step is to more thoroughly analyze the movements of individuals in conjunction with the health data to see if particular movement transitions, e.g., from one region to another at a particular time of year, are associated with changes in health (sensu Figures 7 & 10). Quantifying these transitions may provide operational information to right whale managers by pinpointing vulnerable transition periods in the annual cycle. In addition, there is conjecture that certain sub-classes of the population have fared better than others in times of poor environmental conditions, i.e., the “offshore” animals [27]. Examining this subset with respect to movement and health estimates should sharpen our understanding of how health patterns vary over space and time.

In addition to analyzing movement at the population level, much work remains in examining the health of the population. For example, with this approach we can compare population-wide estimates of health in conjunction with measured vital rates to compare and quantify critical biological processes in the right whale population.

## Conclusion

We have demonstrated a new way to estimate health, movement, and survival of animals over broad spatial and temporal ranges. This represents a fundamental advance in the way we view health of individual animals, and it allows us to integrate many disparate sightings in space and time to generate individual and, ultimately, population estimates of health. This is of paramount importance for right whales – a highly endangered species living in an urban ocean [20], [22] – but in addition the framework can be extended to many different systems. The key foundation of this modeling framework is that it allows for estimates not only of where animals are, but also their health status in each of these areas. This modeling approach offers critical insights into right whale life history and ecology that should enable managers to better make regulatory decisions in order to preserve this species.

## Supporting Information

Appendix S1 **This appendix contains further details on the model and the construction of the Gibbs sampler.** In addition, it details the priors for the ordinal health classes and for movement.
(DOCX)
