# Supplementary material for: Using Hierarchical Bayes to Understand Movement, Health, and Survival in the Endangered North Atlantic Right Whale
Source: PLoS One. 2013 Jun 6;8(6):e64166. doi: 10.1371/journal.pone.0064166 (PMC3675107; doi:10.1371/journal.pone.0064166)
Supplement: Appendix S1 — This appendix contains further details on the model and the construction of the Gibbs sampler. In addition, it details the priors for the ordinal health classes and for movement. (DOCX) [file pone.0064166.s001.docx]

# Using Hierarchical Bayes to understand movement, health, and survival in endangered North Atlantic right whales: Appendix 1

Authors: Robert S. Schick, Scott D. Kraus, Rosalind M. Rolland, Amy R. Knowlton, Philip K. Hamilton, Heather M. Pettis, Robert D. Kenney, and James S. Clark

December 5, 2012

# Modeling Specifics

Here we outline specific details of the model and its various components.

### Data models - Sightings

We will often have zeros contributing to the likelihood, because for any month for which an individual is known or imputed to be alive, but not sighted, it is imputed to be within one of the zones. The random sighting effect associated with individual *i* has the prior

$$gamma\left( \lambda_{i}\left| a,b \right. \right)gamma\left( a\left| a_{1},a_{2} \right. \right)$$

where *b* = 99, and $a_{1}$and $a_{2}$ are specified such that $E\left[ \lambda_{i} \right]=0.01$ is consistent with a prior mean of *λ* = 0.01.

## Data models - Ordinal Health Classes

We constrain the prior for the coefficients in matrix c, and this constraint is implemented at the proposal stage of a Metropolis step (see Computation). Initial slopes *c*_1_*_k_* together with breakpoints are used to initialize matrix *c* as

$$c_{0k}=-\ln\left( \frac{1}{0.5+Q_{k}}-1 \right)-c_{1k}h_{k}^{*}$$

$$Q_{k}=\sum_{j=1}^{k-1} \eta_{j}\left( h_{k}^{*} \right)$$

where $\eta_{j}\left(h_{k}^{*}\right)$ is the probability of class *j* taken at breakpoint *k*.

For missing health status observations, we specify the prior

$$p\left( H_{i,t}^{p}\left| H_{i,t} \right. \right)$$

with the prior vector conditional on truth, rather than vice versa, for coherency and computational convenience (we do not wish to specify a complex relationship $p\left( H_{i,t}\left| H_{i,t}^{p}h_{i,t} \right. \right)$, but rather to solve for it given $p\left( H_{i,t}^{p}\left| H_{i,t} \right. \right)p\left( H_{i,t}\left| h_{i,t} \right. \right)$). Then the model for missing observations is

$$p\left( H_{i,t}\left| H_{i,t}^{p}h_{i,t} \right. \right)=\frac{\eta_{i,t}^{p}\cdot\eta_{i,t}}{\sum_{q=1}^{3} \eta_{i,t,(q)}^{p}\cdot\eta_{i,t,(q)}}$$

where the prior vector depends on the mean health for the individual

$$\eta_{i,t}^{p}=\left\{ \begin{matrix} \left( 0.7, 0.2, 0.1 \right) & \overline{H_{i}}<1.5 \\ \left( 0.2, 0.6, 0.2 \right) & 1.5<\overline{H_{i}}<2.5 \\ \left( 0.1, 0.2, 0.7 \right) & \overline{H_{i}}>2.5 \end{matrix} \right.$$

This formulation indicates that individuals will most often be in a state similar to the states in which they are most often observed.

### Computation

Sighting random effects are sampled from

$$\prod_{t=t_{i}}^{T_{i}} \prod_{k=1}^{K} Pois\left( y_{ik,t}\left| \lambda_{i}E_{k,t} \right. \right)^{u_{ik,t}}gamma\left( \lambda_{i}|a,b \right)=gamma\left( \lambda_{i}\left| a+ \sum_{k,t} y_{i,k,t}, b+ \right.\sum_{k,t} y_{i,k,t}u_{ik,t}E_{k,t} \right).$$

Note that effort is only counted for imputed locations of individual *i* in month *t*. Sampling of the population level sighting intensity *a* is made efficient by integrating out the random effects,

$$\prod_{i=1}^{n} \prod_{t=t_{i}}^{T_{i}} \prod_{k=1}^{K} \left( \int_{0}^{\infty} Pois\left( y_{ik,t}\left| \lambda_{i}E_{k,t} \right. \right)^{u_{ik,t}}gamma\left( \lambda_{i}|a,b \right)d\lambda_{i} \right)\times gamma\left( a\left| a_{1},a_{2} \right. \right)=\prod_{i=1}^{n} \prod_{t=t_{i}}^{T_{i}} \prod_{k=1}^{K} \left( \frac{\Gamma\left( y_{ik,t}+a \right)}{y_{ik,t}!\Gamma\left( a \right)}p_{ik,t}^{y_{ik,t}}\left( 1-p_{ik,t} \right)^{a}gamma\left( a\left| a_{1},a_{2} \right. \right) \right)=\prod_{i=1}^{n} \prod_{t=t_{i}}^{T_{i}} \prod_{k=1}^{K} \left( NB\left( y_{ik,t}\left| a,p_{ik,t} \right. \right) \times gamma\left( a\left| a_{1},a_{2} \right. \right) \right)$$

where

$$p_{ik,t}=\frac{u_{ik,t}E_{k,t}}{b+ u_{ik,t}E_{k,t}}.$$

A Metropolis step is used here.

The latent states for location are sampled conditional on currently imputed previous and next locations (Dupuis 1995; Clark *et al.* 2005). Consider first the case where the individual is imputed to be alive at *t*, because it is imputed be alive at *t*+1, but it was not seen at *t*.

$$\text{Pr}\left( {\left. z_{i,t}=k \right|z}_{i,t}=j, z_{i,t+1}=l \right)=F_{ijl,t}^{-1}f_{ijkl,t}$$

where

$$f_{ijkl,t}=\overset{(t,t-1)}{\overbrace{{\theta_{j,t-1}m}_{kj,t-1}}}\overset{\left( t \right)}{\overbrace{\exp\left( -\lambda_{i}E_{k,t} \right)}}\overset{(t,t+1)}{\overbrace{{\theta_{k,t}m}_{lk,t}}}$$

and

$$F_{ijl,t}^{-1}=\sum_{k=1}^{K} f_{ijkl,t}$$

What the above equation does is put together the different components: survival, movement, and observed/not-observed over the three time intervals. The terms under the left-most brace show the probability the animal survived and made the move from *j* to *k*. The term under the middle brace shows the sighting probability. Finally, the term under the right brace shows survival over the next time period along with movement from *k* to *l*.

If *i* is currently imputed to be alive at *t* and to have died in region *l* on the interval (*t*, *t*+1), we have the probability that it is still alive and in *k* at time *t*

$$\text{Pr}\left( z_{i,t}=k, s_{ik,t-1}=1\left| z_{i,t-1}=j, z_{i,t+1}=l, s_{ik,t}=0 \right. \right)=G_{ijl,t}^{-1}\text{g}_{1,ijkl,t}$$

$$\text{g}_{1,ijkl,t}=\theta_{j,t-1}m_{kj,t-1}\text{exp}\left( -\lambda_{i}E_{k,t} \right)\left( 1-\theta_{k,t} \right)m_{lk,t}$$

and the probability that it dies on the interval (*t*-1,*t*) in k

$$\text{Pr}\left( z_{i,t}=k, s_{ik,t-1}=0\left| z_{i,t-1}=j, z_{i,t+1}=l, s_{ik,t}=0 \right. \right)=G_{ijl,t}^{-1}\text{g}_{0,ijkl,t}$$

$$\text{g}_{0,ijkl,t}=\left( 1-\theta_{j,t-1} \right)m_{kj,t-1}$$

where

$$G_{ijl,t}^{-1}=\sum_{s=0}^{1} \sum_{k=1}^{K} \text{g}_{s,ijkl,t}$$

If *t* is the last census there is no conditioning on the following time

$$\text{Pr}\left( z_{i,t}=k, s_{ik,t-1}=1\left| z_{i,t-1}=j \right. \right)=H_{ijl,t}^{-1}h_{ijkl,t}$$

$$h_{1ijkl,t}= \theta_{j,t-1}m_{kj,t-1}\text{exp}\left( -\lambda_{i}E_{k,t} \right)$$

$$h_{0ijkl,t}=\text{g}_{0,ijkl,t}$$

$$H_{ijl,t}^{-1}=\sum_{s=0}^{1} \sum_{k=1}^{K} \text{h}_{s,ijkl,t}$$

If it was last sighted or imputed to be alive at t-1, and it is imputed to be dead at t+1, then it could have died on the interval (t-1, t),

$$\text{Pr}\left( s_{ij,t}=0\left| z_{i,t-1}=j \right. \right)=1- \theta_{j,t-1}$$

or survived to t, moved to location k, and then died with probability

$$\text{Pr}\left( z_{i,t}=k, s_{ik,t+1}=0|z_{i,t-1}=j, s_{ik,t+1}=0 \right)=C^{-1}m_{kj,t-1}\theta_{j,t-1}exp\left( -\lambda_{i}E_{k,t} \right)\left( 1-\theta_{k,t} \right)$$

with the normalizer

$$C= \theta_{j,t-1}\sum_{k=1}^{K} m_{kj,t-1}exp(-\lambda_{i}E_{k,t})(1-\theta_{k,t})$$

Movement is sampled from

$$m_{j,t^{'}}\sim Dir\left( q_{j,t^{'}}+\sum_{i,t\in t^{'}} ,w_{ikj,t} \right)$$

where m*_j,t'_* is movement in month of the year *t'*, and the prior $Dir\left( m_{j,t^{'}}|q_{j,t^{i}} \right)$ is specified by gender (not subscripted) and month *t'*.

Survival is Metropolis, with parameters initialized at $\beta=\left( X^{T}X \right)^{-1}X^{T}Z$

where *X* are the stacked vectors *x_ik,t_* and *Z* is the corresponding stacked vector of logit(*z_i,t_*), where *z_i,t_* = 0.1 if *i* known to be alive at *t*, and *z_i,t_* = 0.9 otherwise. Proposals are from

$$N\left( \beta_{\left( g \right)},\left( X^{T}X \right)^{-1} \right)$$

where the mean vector is the current estimate for Gibbs step *g*. For the parameters of this Normal distribution, we follow Clark 2007 to generate *V* and *v*, where *Vv* is the mean and *V* is the variance. Hence $Vv=\left( X^{T}X \right)^{-1}X^{T}Z$ is the mean and $V=\left( X^{T}X \right)^{-1}$ is the variance.

Health status is Metropolis, sampled from

$$N\left( h_{i,t}|w_{i,t-1}\alpha, \sigma^{2} \right)N\left( h_{i,t+1}|w_{i,t}\alpha,\sigma^{2} \right)multinom\left( H_{i,t}|\eta_{i,t} \right)\theta_{ik,t}^{s_{ik,t}}\left( 1-\theta_{ik,t} \right)^{1-s_{ik,t}}=N\left( h_{i,t}|Vv, V \right)multinom\left( H_{i,t}|\eta_{i,t} \right)\theta_{ik,t}^{s_{ik,t}}\left( 1-\theta_{ik,t} \right)^{1-s_{ik,t}}$$

where

$$V^{-1}=\frac{1}{\sigma^{2}}I\left( a_{i,t-1}>0 \right)+\frac{\alpha_{2}^{2}}{\sigma^{2}}I\left( a_{i,t+1}<A_{i} \right)$$

$$v= \frac{w_{i,t-1}\alpha}{\sigma^{2}}I\left( a_{i,t-1}>0 \right)+\frac{\left( h_{i,t+1}-w_{i,t\left( -2 \right)}\alpha_{\left( -2 \right)} \right)}{\sigma^{2}}I\left( a_{i,t+1}<A_{i} \right)$$

where the indicator functions include terms for transitions where the individual was already alive at *t*-1, $I\left( a_{i,t-1}>0 \right)$ but not yet imputed to be dead at *t*+1, $I\left( a_{i,t+1}<A_{i} \right)$.

Health status parameters are sampled from

$$N\left( \alpha|Vv, V \right)$$

$$V^{-1}=\frac{1}{\sigma^{2}}W^{T}W+V_{\alpha}^{-1}$$

$$v= \frac{1}{\sigma^{2}}W^{T}h+V_{\alpha}^{-1}\mu_{\alpha}$$

where *W* is the stacked matrix of *w_i,t_*_-1_, *h* is the stacked vector *h_i,t_*, *V_a_* is the prior covariance matrix, and *μ_a_* is the prior mean vector.

The process variance has an informative prior and is sampled directly from

$$IG\left( \sigma^{2}|h_{1}+0.5\sum_{i=1}^{n} \left( T_{i}-t_{i} \right), h_{2}+0.5\sum_{i=1}^{n} \sum_{t=t_{i}+1}^{T_{i}} \left( h_{i,t}-x_{i,t-1}\beta\right)^{2} \right)$$

where *T_i_* is the year in which the individual is imputed to have died or was censored, and prior parameter values are *h*_1_ = *n*×*T*, *h*_2_ = 5^2^(*h*_1_ - 1), where *T* is the total duration of the study. This informative prior carries weight roughly equivalent to the data and has a mean standard deviation of *h* = 5.

Missing health status observations are sampled from

$$p\left( H_{i,t}\left| H_{i,t}^{p}h_{i,t} \right. \right)=\frac{\eta_{i,t}^{p}\cdot\eta_{i,t}}{\sum_{q=1}^{3} \eta_{i,t,(q)}^{p}\cdot\eta_{i,t,(q)}}$$

Health status observation parameters are sampled using a Metropolis step with the inequality imposed at the proposal stage,

$$multinom\left( H_{i,t}|\eta_{i,t} \right)N\left( c|\mu_{c}, V_{c} \right)I\left( c_{01}<c_{02}, c_{11}<c_{12} \right)$$

# Priors for Health

Here we show the informed priors we used for the links between photographic observations of health status, *H*, and true health *h* (See Methods section in main text). Note that priors for these govern the midpoint of the likely transition from one health class to another, along with the steepness of the transition. Figure S1 depicts the prior breakpoints between the discrete health classes that were used in the model.

The priors for body fat and entanglement status are very similar. Note the transition between class 1 and 2 in each case is steeper than the transition between class 2 and 3. This reflects prior belief that animals observed in the lowest category, i.e. 1, are clearly in poor health, whereas the transitions between the healthier classes are less certain.

Because of the potentially broad range of skin conditions in class 1 (see Discussion in main text), we have assumed a much broader interval over which the transitions could occur and much more gradual transitions between these classes.

Figure S 1. Graphical description of the quantitative relationship between health status on the latent scale (x axis), and the probability of an animal being seen and categorized in a discrete health class for each of the 6 different observation categories of health. Grey boxes indicate the range over which the transition from one class to another could occur; lines depict the probability of being observed in a class given your position on the true health scale. Text markings refer to the name of the class, i.e. class 1, class 2, etc.

# Priors on Movement

Below we show the priors as narrative belief of the transition between geographic regions at specific times. The priors are for each gender: male, female, and unknown for each of 12 timesteps. Here is an example of the narrative statement for females in January.

January

- Adult females:
  - 23% from SEUS to SEUS,
  - 5% from MIDA to SEUS,
  - 10% from unknown to SEUS,
  - 10% from GoM to NE,
  - 2% from NE to NE,
  - 3% from NE to MIDA,
  - 3% from unknown to NE,
  - 5% from GoM to GSC.

Note that we have 81 possible transitions for the 9 regions. For the transitions where we have some prior belief, we will populate a 9 * 9 matrix with the above percentages as follows. For SEUS, we have three transitions into SEUS in January, from SEUS, from MIDA, and from unknown. In this case, we’ll populate the SEUS to SEUS transition with 0.23, MIDA to SEUS with 0.05, and the remaining 7 transitions with 0.014.

However, the priors for movement are based on weights, not percentages (See Methods section in main text). Accordingly, we took two more steps to generate the prior weights. First we scaled the above by dividing by the column sum. Then we scaled these values as follows:

$$x=wt*\frac{\left( x-\min\left( x \right) \right)}{\max\left( x \right)-\min\left( x \right)}+1$$

where x is the 9 by 9 matrix of prior movement probabilities, and wt is the scalar (in this case *wt =* n, i.e. *wt* = 622). The final prior matrix for the transitions between and among regions for females in one region in January moving to another region in February highlights these weights (Figure S2).


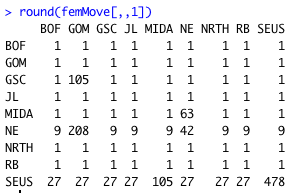


Figure S 2. Prior weights for the transitions from one region to another in January to February for adult females. Higher numbers indicate prior belief that the transition will occur.

Below are all of the narrative priors for movement for all transitions for all population segments over all months. Note that while juveniles are included below, currently we are only modeling different gender classes, not age and gender.

## Narrative Summary

January

- Adult females:
  - 23% from SEUS to SEUS,
  - 5% from MIDA to SEUS,
  - 10% from unknown to SEUS,
  - 10% from GoM to NE,
  - 2% from NE to NE,
  - 3% from NE to MIDA,
  - 3% from unknown to NE,
  - 5% from GoM to GSC.
- Juvenile females:
  - 3% from GoM to GoM,
  - 5% from unknown to GSC,
  - 3% from unknown to MIDA,
  - 35% from SEUS to SEUS,
  - 7% from MIDA to SEUS,
  - 2% from NE to NE,
  - 3% from unknown to NE.
- Adult males:
  - 3% from MIDA to SEUS,
  - 8% from GSC to MIDA,
  - 10% from GOM to SEUS,
  - 4% GOM to GSC,
  - 10% GoM to GoM,
  - 2% JL to JL,
  - 2% from unknown to JL,
  - 8% unknown to NE.
- Juvenile males:
  - 3% from GoM to GoM,
  - 5% from unknown to GSC,
  - 3% from unknown to MIDA,
  - 35% from SEUS to SEUS,
  - 7% from MIDA to SEUS,
  - 2% from NE to NE,
  - 3% from unknown to NE.

February

- Adult females:
  - 5% from GoM to GoM,
  - 5% from GSC to GSC,
  - 3% from GoM to GSC,
  - 15% from SEUS to MIDA,
  - 10% from SEUS to NE,
  - 2% from MIDA to MIDA,
  - 2% from unknown to MIDA,
  - 10% from SEUS to SEUS,
  - 5% from MIDA to SEUS,
  - 5% from unknown to SEUS,
  - 10% from NE to NE,
  - 3% from unknown to NE.
- Juvenile females:
  - 7% from SEUS to NE,
  - 3% from MIDA to NE,
  - 18% from SEUS to SEUS,
  - 4% from unknown to SEUS,
  - 4% from SEUS to MIDA,
  - 5% from unknown to NE,
  - 5% from GSC to GSC,
  - 3% from unknown to GSC.
- Adult males:
  - 2% GoM to Gom,
  - 5% GSC to GSC,
  - 3% GoM to GSC,
  - 2% GoM to MIDA,
  - 2% from unknown to MIDA,
  - 2% GoM to SEUS,
  - 5% SEUS to SEUS,
  - 3% from MIDA to SEUS,
  - 5% from unknown to SEUS,
  - 5% from NE to NE,
  - 15% from unknown to NE,
  - 7% from SEUS to NE,
  - 3% from unknown to GoM,
  - 3% from unknown to GSC.
- Juvenile males:
  - 5% from SEUS to NE,
  - 3% from MIDA to NE,
  - 20% from SEUS to SEUS,
  - 5% from MIDA to SEUS,
  - 2% from SEUS to MIDA,
  - 10% from unknown to NE,
  - 5% from GSC to GSC,
  - 3% from unknown to GSC.

March

- Adult females:
  - 15% from unknown to NE,
  - 15% from SEUS to NE,
  - 10% from MIDA to NE,
  - 2% from SEUS to SEUS,
  - 5% from SEUS to MIDA,
  - 5% from SEUS to GSC,
  - 5% from unknown to GoM,
  - 3% from NE to GSC,
  - 5% from unknown to GSC,
  - 3% from GSC to GSC,
  - 2% from unknown to JL.
- Juvenile females:
  - 10% from GSC to GSC,
  - 5% from unknown to GSC,
  - 10% from NE to NE,
  - 20% from SEUS to NE,
  - 2% from unknown to GoM,
  - 5% from NE to GSC.
- Adult males:
  - 3% from GoM to GoM,
  - 3% from GoM to GSC,
  - 6% from GSC to GSC,
  - 15% from unknown to NE,
  - 10% from NE to NE,
  - 2% from SEUS to MIDA,
  - 10% from unknown to GSC.
- Juvenile males:
  - 10% from GSC to GSC,
  - 5% from unknown to GSC,
  - 10% from NE to NE,
  - 20% from SEUS to NE,
  - 2% from unknown to GoM,
  - 5% from NE to GSC.

April

- Adult females:
  - 2% from SEUS to GSC,
  - 8% from NE to NE,
  - 20% from NE to GSC,
  - 5% from NE to GoM,
  - 1% from SEUS to MIDA,
  - 3% from unknown to GoM,
  - 15% from unknown to GSC.
- Juvenile females:
  - 5% from GSC to GSC,
  - 2% from SEUS to GSC,
  - 15% from unknown to GSC,
  - 12% from NE to GSC,
  - 3% from unknown to GoM,
  - 6% from NE to NE.
- Adult males:
  - 8% from NE to NE,
  - 20% from NE to GSC,
  - 18% from unknown to GSC;
  - 5% from GoM to GoM,
  - 13% from unknown to GoM,
  - 10% from GSC to GSC.
- Juvenile males:
  - 5% from GSC to GSC,
  - 2% from SEUS to GSC,
  - 15% from unknown to GSC,
  - 12% from NE to GSC,
  - 3% from unknown to GoM,
  - 6% from NE to NE.

May

- Adult females:
  - 30% from GSC to GSC,
  - 8% from GoM to GoM,
  - 12% from GSC to GoM,
  - 5% from GoM to GSC,
  - 5% from unknown to NE,
  - 5% from NE to NE,
  - 25% from GSC to unknown,
  - 2% from unknown to JL,
  - 5% from unknown to BOF,
  - 5% from unknown to GSC,
  - 1% from GSC to NRTH.
- Juvenile females:
  - 8% from GoM to GoM,
  - 15% from GSC to GSC,
  - 8% from GSC to GoM,
  - 4% from unknown to GoM,
  - 15% from GSC to unknown,
  - 7% from unknown to GSC.
- Adult males:
  - 30% from GSC to GSC,
  - 12% from GoM to GoM,
  - 20% from GSC to GoM,
  - 2% from GoM to GSC,
  - 25% from GSC to unknown.
- Juvenile males:
  - 8% from GoM to GoM,
  - 15% from GSC to GSC,
  - 8% from GSC to GoM,
  - 4% from unknown to GoM,
  - 15% from GSC to unknown,
  - 7% from unknown to GSC.

June

- Adult females:
  - 5% from GSC to BoF,
  - 2% from GoM to RB,
  - 10% from GoM to BoF,
  - 4% from unknown to BoF,
  - 10% from GSC to GoM,
  - 10% from GSC to unknown,
  - 5% from GSC to NRTH,
  - 5% from NE to NE,
  - 5% from unknown to JL,
  - 5% from GSC to GSC,
  - 8% from GoM to GoM,
  - 5% from GoM to JL,
  - 5% from unknown to GSC.
- Juvenile females:
  - 3% from NE to NE,
  - 8% from GoM to BoF,
  - 3% from GoM to GoM,
  - 6% from GoM to unknown,
  - 6% from GSC to unknown,
  - 3% from GSC to GSC.
- Adult males:
  - 3% from GoM to RB,
  - 1% from GSC to BoF,
  - 2% from GoM to BoF,
  - 1% from unknown to BoF,
  - 10% from GSC to GoM,
  - 10% from GSC to unknown,
  - 5% from NE to NE,
  - 5% from unknown to JL,
  - 5% from GSC to GSC,
  - 8% from GoM to GoM,
  - 5% from GoM to JL,
  - 5% from unknown to GSC,
  - 2% from unknown to NRTH.
- Juvenile males:
  - 3% from NE to NE,
  - 8% from GoM to BoF,
  - 3% from GoM to GoM,
  - 6% from GoM to unknown,
  - 6% from GSC to unknown,
  - 3% from GSC to GSC.

July

- Adult females:
  - 3% from GoM to NRTH,
  - 25% from GoM to BoF,
  - 2% from GoM to GoM,
  - 2% from GSC to GSC,
  - 2% from unknown to JL,
  - 3% from BoF to BoF,
  - 2% from unknown to NRTH,
  - 2% from BoF to NRTH,
  - 1% from NE to NE,
  - 1% from unknown to NE,
  - 2% from GoM to RB.
- Juvenile females:
  - 3% from unknown to RB,
  - 2% from unknown to NE,
  - 10% from GoM to BoF,
  - 15% from unknown to BoF,
  - 4% from BoF to BoF.
- Adult males:
  - 25% from GoM to BoF,
  - 2% from GoM to GoM,
  - 10% from unknown to GoM,
  - 3% from GSC to GSC,
  - 15% from GoM to RB,
  - 1% from NE to NE,
  - 1% from unknown to NE,
  - 4% from Unknown to NRTH.
- Juvenile males:
  - 4.5% from unknown to RB,
  - 2% from unknown to NE,
  - 10% from GoM to BoF,
  - 15% from unknown to BoF,
  - 4% from BoF to BoF,

August

- Adult females:
  - 30% from BoF to BoF,
  - 3% from RB to BoF,
  - 7% from unknown to BoF,
  - 5% from BoF to RB,
  - 2% from NRTH to NRTH,
  - 2% from unknown to NRTH,
  - 2% from BoF to JL,
  - 2% from NE to NE,
  - 2% from unknown to NE,
  - 3% from unknown to GoM,
  - 1% from unknown to JL.
- Juvenile females:
  - 35% from BoF to BoF,
  - 10% from unknown to BoF,
  - 5% from unknown to RB.
- Adult males:
  - 20% from BoF to BoF,
  - 15% from BoF to RB,
  - 5% from unknown to RB,
  - 10% from RB to BoF,
  - 5% from unknown to BoF,
  - 2% from unknown to NRTH,
  - 2% from BoF to JL,
  - 2% from NE to NE,
  - 2% from unknown to NE,
  - 3% from unknown to GoM,
  - 1% from unknown to JL.
- Juvenile males:
  - 35% from BoF to BoF,
  - 10% from unknown to BoF,
  - 8% from unknown to RB,
  - 8% from BoF to RB.

September

- Adult females:
  - 5% from BoF to BoF,
  - 2% from unknown to BoF,
  - 2% from unknown to RB,
  - 2% from BoF to RB,
  - 5% from BoF to GoM,
  - 2% from unknown to GoM,
  - 5% from BoF to JL,
  - 6% from unknown to JL.
- Juvenile females:
  - 5% from BoF to BoF,
  - 2% from unknown to BoF,
  - 2% from unknown to RB,
  - 5% from BoF to GoM,
  - 2% from unknown to GoM,
  - 5% from BoF to JL,
  - 6% from unknown to JL.
- Adult males:
  - 6% from BoF to BoF,
  - 2% from unknown to BoF,
  - 8% from BoF to RB,
  - 2% from unknown to RB,
  - 5% from BoF to GoM,
  - 2% from unknown to GoM,
  - 10% from BoF to JL,
  - 15% from unknown to JL.
- Juvenile males:
  - 5% from BoF to BoF,
  - 2% from unknown to BoF,
  - 2% from unknown to RB,
  - 5% from BoF to GoM,
  - 2% from unknown to GoM,
  - 5% from BoF to JL,
  - 6% from unknown to JL.

October

- Adult females:
  - 2% from NRTH to MIDA,
  - 10 from unknown to GoM,
  - 5% from GoM to JL,
  - 10% from unknown to JL,
  - 2% from unknown to MIDA,
  - 2% from unknown to NE,
  - 1% from unknown to SEUS,
  - 4% from unknown to BoF.
- Juvenile females:
  - 10 from unknown to GoM,
  - 3% from GoM to JL,
  - 6% from unknown to JL,
  - 1% from unknown to MIDA,
  - 2% from unknown to NE,
  - 4% from unknown to BoF.
- Adult males:
  - 15 from unknown to GoM,
  - 10% from GoM to JL,
  - 15% from unknown to JL,
    2% from unknown to MIDA,
  - 2% from unknown to NE,
  - 1% from unknown to SEUS,
  - 4% from unknown to BoF.
- Juvenile males:
  - 10 from unknown to GoM,
  - 3% from GoM to JL,
  - 9% from unknown to JL,
  - 1% from unknown to MIDA,
  - 2% from unknown to NE,
  - 4% from unknown to BoF.

November

- Adult females:
  - 5% from MIDA to SEUS,
  - 10% from unknown to SEUS,
  - 4% from unknown to JL,
  - 3% from GoM to JL,
  - 2% from JL to JL,
  - 2% from GoM to GoM,
  - 2% from unknown to GoM,
  - 10% from unknown to MIDA,
  - 1% from unknown to BoF,
  - 2% from NRTH to SEUS.
- Juvenile females:
  - 5% from MIDA to SEUS,
  - 10% from unknown to SEUS,
  - 20% from GoM to SEUS,
  - 2% from unknown to JL,
  - 1% from GoM to JL,
  - 1% from JL to JL,
  - 2% from unknown to GoM,
  - 10% from unknown to MIDA,
  - 1% from unknown to BoF.
- Adult males:
  - 1% from unknown to SEUS,
  - 1% from unknown to MIDA,
  - 10% from GoM to GoM,
  - 15% from unknown to GoM,
  - 3% from JL to GoM,
  - 5% from JL to JL,
  - 7% from unknown to JL,
  - 1% from unknown to BoF.
- Juvenile males:
  - 5% from MIDA to SEUS,
  - 10% from unknown to SEUS,
  - 20% from GoM to SEUS,
  - 2% from unknown to JL,
  - 1% from GoM to JL,
  - 1% from JL to JL,
  - 2% from unknown to GoM,
  - 10% from unknown to MIDA,
  - 1% from unknown to BoF.

December-

- Adult females:
  - 5% from unknown to SEUS,
  - 12% from GoM to the SEUS,
  - 5% from SEUS to SEUS,
  - 7% from unknown areas to NE,
  - 5% from GoM to GoM,
  - 2% from NE to MIDA,
  - 4% from unknown to MIDA,
  - 5% from MIDA to SEUS,
  - 4% from unknown to GoM,
  - 2% from GoM to JL,
  - 2% from NRTH to SEUS.
- Juvenile females:
  - 45% of juvenile females from SEUS to SEUS,
  - 10% from MIDA to SEUS,
  - 10% from NE to SEUS.
- Adult males:
  - 25% from GoM to GoM,
  - 5% from GoM to SEUS,
  - 2% from GoM to MIDA,
  - 2% from GoM to NE,
  - 2% from GoM to JL,
  - 2% JL to JL
- Juvenile males:
  - 45% of juvenile males from SEUS to SEUS,
  - 10% from MIDA to SEUS,
  - 10% from NE to SEUS.
